# Comparison of the Antimicrobial Efficacy of Various Contact Lens Solutions to Inhibit the Growth of *Pseudomonas aeruginosa* and *Staphylococcus aureus*

**DOI:** 10.1155/2018/5916712

**Published:** 2018-05-31

**Authors:** B. Laxmi Narayana, Pooja Rao, Sevitha Bhat, K. Vidyalakshmi

**Affiliations:** Department of Microbiology, Kasturba Medical College, Manipal Academy Higher Education, Mangalore, Karnataka 575001, India

## Abstract

Soft contact lenses provide perfect conditions for the breeding of pathogens. The study is a prospective, experimental study, conducted to know the antimicrobial ability of multipurpose contact lens solution against standard strains of *Staphylococcus aureus* and *Pseudomonas aeruginosa* by the stand-alone test. The test method is based on the procedures in the ISO 14729 standard primary stand-alone test. Three multipurpose contact lens care solutions commercially available in Mangalore markets, namely, Biotrue (Bausch & Lomb), Opti-Free Replenish (Alcon), and Aquasoft (Stericon Pharma), were tested for its antimicrobial effect in the microbiology lab at Kasturba Medical College, Mangalore. According to this study, the solutions named “Biotrue” and “Aquasoft” met the primary stand-alone and reached the 3log reduction and 5log reduction criteria in the manufacturer recommended time, respectively. No conclusion could be drawn for Opti-Free Replenish since the minimum recommended disinfection time was overnight, whereas it was noted for 6 hr only, and it should have been experimented further. The effectiveness of multipurpose solutions varies against different bacterial species. We have observed that the antimicrobial activity of different solutions varies with respect to time of incubation, and also there was a marked difference in the activity of some solutions against *S. aureus* and *P. aeruginosa*. So, it is necessary for the contact lens users to store their lenses in solutions for longer duration of hours. It is also recommended to use solutions that clear the ISO 14729 standards for better health conditions of the eye.

## 1. Introduction

Diseases related to the eye are common and frequently seen in clinical practice. Soft contact lenses provide perfect conditions for the breeding of pathogens. Therefore, disinfecting solutions for contact lenses are of utmost importance. The solutions must be effective in inhibiting the growth of pathogens and protecting its users from infections [1].

Different pathogens such as *Staphylococcus aureus*, *Pseudomonas aeruginosa, Escherichia coli*, and other organisms commonly cause several eye infections such as bacterial keratitis, bacterial conjunctivitis, and so on [2]. Contact lens solutions may harbour a good number of organisms due to unhygienic practices by the users; climatic changes especially humidity may become one of the contributing factors. Excessive contact lens wearers are more prone to infections and require safety concerns. Because of the emergence of virulence factors, contact lens solutions have a reduced ability to inhibit their growth [1]. Some investigations have shown that contact lens-related keratitis is commonly caused by bacteria, such as *Staphylococcus aureus* and *Pseudomonas aeruginosa* [3]. Clinical practitioners expose their patients to contact lens solutions by recommending certain brands for cleaning and disinfecting of their contact lenses. Therefore, clinicians need to be aware of how the solutions are susceptible to the growth of microorganisms [1]. Hence, this study will help the clinicians and users to know the efficacy of some of the multipurpose contact lens solutions.

## 2. Materials and Methods

The three multipurpose contact lens solutions (MPS) commercially available in the market were tested for their antimicrobial effect in the microbiology lab at KMC, Mangalore. It is a prospective, experimental, comparative study. Standard strains of *Staphylococcus aureus* ATCC 25923 and *Pseudomonas aeruginosa* ATCC 27853 were used for this test. Three multipurpose contact lens care solutions commercially available in Mangalore markets, namely, Biotrue (Bausch & Lomb), Opti-Free Replenish (Alcon), and Aquasoft (Stericon Pharma), were evaluated. Normal saline was used as a control with each batch. The products were well within expiry date and tested according to the manufacturer labeled instructions for minimum disinfection time. The test method was based on the procedures in the ISO 14729 standard primary stand-alone test.

The antimicrobial efficacy of the regimen was evaluated according to the ISO 14729 standard required for the primary stand-alone test. A total of 40 test tubes were used for evaluation of three solutions plus one control solution (sterile saline). Two test tubes prepared for each of three solutions were tested for a time interval of 0 min, 10 min, 30 min, 2 hr, 4 hr, and 6 hr. The 40 test tubes were marked as solution A, B, C, and control to prevent bias. The inoculum prepared with 1 ml of the 0.5 McFarland standard preparation (1.5 ∗ 10^8^ CFU/ml) was added to 2 ml of sterile saline/BHI (1 : 3), and then 1 ml of this was added to 9 ml of sterile saline to give 1 : 10. The final prepared suspension in test tubes was made to 2 ml, consisting of 1900 *µ*l of solution A, B, C, or control solution and 100 *µ*l of the inoculum. After this, the suspensions were incubated at specific time intervals, and 100 *µ*l of each prepared inoculum was plated onto the MacConkey's agar plates and incubated overnight at 35°C in the ambient air and then followed by colony count and calculation of mean for each suspension per time interval. The determination of the logarithmic reduction of the growth in each solution and the control was calculated by the following equation:

(1) $$\begin{array}{c} \log\text{ reduction}=\log10\left(\text{initial }\text{CFU}/\text{ml}\right)-\log10\left(\text{final }\text{CFU}/\text{ml}\right). \end{array}$$

The study has received approval from the institutional ethics committee.

## 3. Results

According to the primary stand-alone criteria, the tested MPS should reduce the bacterial colonies by 3log units. The positive control had 10^5^ colonies on plating onto the media. Colony counts per solution per time interval for *S. aureus and P. aeruginosa* are shown in Tables 1 and 2. The mean logarithmic reduction in the minimum recommended disinfection time for each of the MPS against the standard *S. aureus* strain (ATCC 25923) and standard *P. aeruginosa* strain (ATCC 27853) is shown in Tables 3 and 4, respectively.

**Figure 1:**
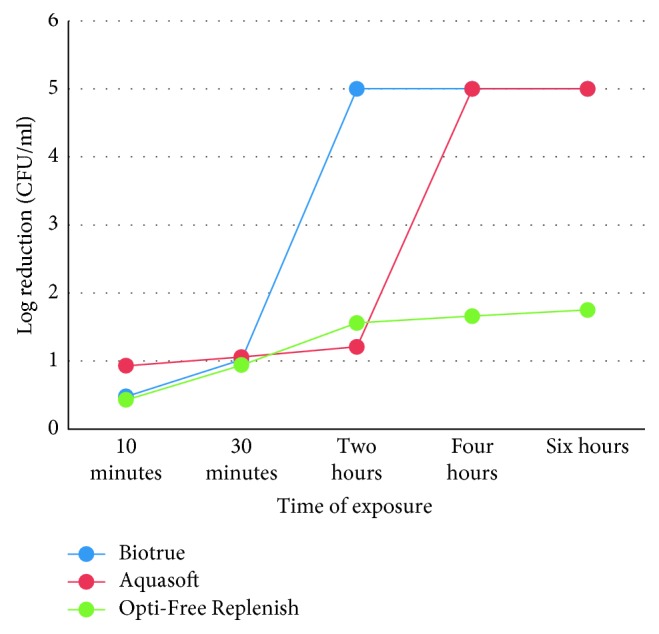
Effects of different MPS and length of exposure on the concentration of *Staphylococcus aureus*.

There was a great variation in the effects of different MPS on *Staphylococcus aureus*. Biotrue showed good activity towards *S. aureus* compared with other solutions (Figure 1).

**Figure 2:**
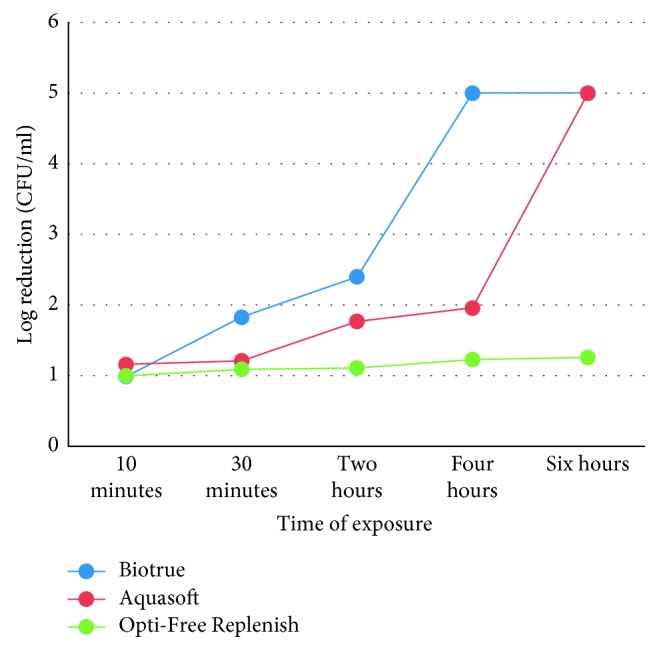
Effects of different MPS and length of exposure on the concentration of *Pseudomonas aeruginosa.*

There was a great variation in the effects of different MPS on *Pseudomonas aeruginosa*. Biotrue showed good activity towards *Pseudomonas* compared with other solutions (Figure 2).

Aquasoft passed the ISO 14729 primary stand-alone test after 4 hr on *S. aureus*, but in the case of *P. aeruginosa*, it has passed the test in 6 hr. It is to be noted that there is no use of contact lens in this experiment; hence, the action of the solutions may increase if the contact lens is rubbed or rinsed in the solution. On *S. aureus*, Aquasoft showed 5log reduction in 4 hr and on *P. aeruginosa* in 6 hr. Since the solution passed primary stand-alone criteria for both organisms in the recommended time, the requirement for subjecting it to the second test was ruled out. Biotrue passed the ISO 14729 primary stand-alone test within the manufacturer recommended disinfection time (4 hr). *S. aureus* showed 5log reduction after 2 hr, whereas *P. aeruginosa* showed after 4 hr. No conclusion could be drawn for Opti-Free Replenish since the minimum recommended disinfection time was at least 6 hr or overnight, whereas it was noted for 6 hr only. It showed log reduction of 1.75 on *S. aureus* and 1.26 on *P. aeruginosa*. However, the manufacturer guidelines say that soaking the lens overnight in the solution will show 3log reduction.

In the present study (ISO 14729 primary stand-alone test), Biotrue has shown good antimicrobial activity as compared to Aquasoft and Opti-Free Replenish. In the case of Biotrue, *S. aureus* reached the 3log reduction criteria within 2 hr of incubation, whereas *P. aeruginosa* reached in 4 hr. Similar results were found with the Aquasoft solution too, the 5log reduction after 4 hr of incubation for *S. aureus* and 6 hr for *P. aeruginosa*.

## 4. Discussion

Contact lenses are easily susceptible to the microbial contamination, so it is necessary for the contact lens solutions to meet the ISO 14729 standards for the stand-alone test. Noncompliance is one of the factors that commonly lead to the contact lens-related microbial keratitis [4, 5]. In the present study, we tested three different contact lens disinfecting solutions, of which Biotrue chemically contains polyaminopropyl biguanide (0.00013%) and polyquaternium (0.0001%), Opti-Free Replenish contains polyquaternium/Polyquad (0.001%) and myristamidopropyl dimethylamine/Aldox (0.0005%), and Aquasoft contains polyhexamide. Polyhexamide is a disinfectant which carries highly charged active sites that can disrupt microbial cellular membranes by electrostatic interaction which are most effective against a wide range of microorganisms [6, 7]. Polyquaternium (Polyquad) is a quaternary ammonium-based antimicrobial agent and has antibacterial properties, while MAPD showed a wide spectrum of antimicrobial activity on fungi which is not tested in our study. No previous study was done with Biotrue (Bausch & Lomb) for *S. aureus* and *P. aeruginosa*, but in one of the studies conducted by Lever and Roya, Renu multipurpose solution which is manufactured by the same manufacturer with similar disinfecting agents, that is, polyaminopropyl biguanide (0.0001%), showed the mean log reduction of >4.6 for *S. aureus* and >4.2 for *P. aeruginosa*, and it has achieved the minimum disinfection criteria more quickly over other tested solutions [8]. According to the study done by Mohammadinia et al., Renu multipurpose solution failed to meet the ISO stand-alone criteria for *Pseudomonas aeruginosa* [3]. In the present study, Biotrue showed a significantly higher disinfecting property against *S. aureus*, and for *P. aeruginosa*, the mean reduction of 5 was seen in *S. aureus* (2 hr) and *P. aeruginosa* (4 hr), respectively.

In the present study, Opti-Free Replenish which contains 0.001% polyquaternium (Polyquad) and 0.0005% Aldox failed to meet 3log reduction in 6 hr for *S. aureus* and *P. aeruginosa*. In 6 hr, *S. aureus* and *P. aeruginosa* have shown a mean reduction of 1.75 and 1.26, respectively. However, it should be noted that the product literature says “store lenses in the closed lens case overnight or at least 6 hours.” One of the earlier studies conducted by Marsha et al., Opti-Free Express which is similar to Opti-Free Replenish with respect to disinfectants did not show 3log reduction within 6 hr of incubation, where *S. aureus* showed the mean reduction of 1.252 in 6 hr. It is notable that their studies mention for a re-evaluation of the said solution with the secondary regimen test [1]. Similar findings were observed in studies of Lever and Roya too [8].

Aquasoft gained the 3log reduction criteria within 4 hr for *S. aureus* and 6 hr for *P. aeruginosa* having the log reduction of 5. To the best of our knowledge, there are no previous studies which were conducted with this particular solution. One of the previous studies of Iguban et al. using Solocare, where the same polyhexamide was used as the disinfecting agent, also showed good antimicrobial activity against *P. aeruginosa* and *C. albicans* [9].

The study conducted by Sakuma et al. observed that none of their MPS was good enough to remove the organism by the primary stand-alone test alone and patients should follow standard instructions carefully, including the cleaning and rinsing of contact lenses. There are other factors which can eliminate the number of organisms; it should be kept in mind that complete antimicrobial activity can be attained only when there is a combination of cleaning, rinsing, and disinfecting the lenses [10]. All the study solutions reached 3log reduction, respectively, in the manufacturer recommended time except Opti-Free Replenish because it needs to be tested for overnight incubation (8 hr) to pass the stand-alone criteria. The observed percentage reduction for Opti-Free Replenish at 6 hr for *S. aureus* and *P. aeruginosa* was 98.1% and 94.4%, respectively. For 6-hour duration, the solution has not passed the required criteria, which is also one of the recommendations according to the literature. Probably overnight incubation would have passed the stand-alone test which was not done in this study. In comparison to “Unique A,” Opti-Free Replenish which is composed of Polyquad similar to the former showed less antibacterial activity, while according to the studies conducted by Kuzman et al., “Unique A” showed excellent activity on *S. aureus* and *P. aeruginosa* [11]. According to Hildebrandt et al., Opti-Free Replenish with organic load passed the stand-alone test criteria [12]. It has reached the 3log reduction for *S. aureus* and *P. aeruginosa*. But in the absence of organic load, it had shown the mean log reduction less than 3log, and it had failed the stand-alone criteria. These findings are comparable with our results [10].

According to the study of Mohammadinia et al., the clinically isolated strains are more resistant than standard strains [3]. For virulent strains, MPS takes longer incubation time for their antimicrobial action, so it is recommended that further study is required to determine the effectiveness of MPS against such organisms which is one of the limitations of our study. In the present study, we can analyze that the solutions which containing polyaminopropyl biguanide (0.00013%), polyquaternium (0.0001%) and polyquaternium/Polyquad (0.001%), and myristamidopropyl dimethylamine/Aldox (0.0005%) have good disinfecting property against *S. aureus* and *Pseudomonas aeruginosa* but polyhexamide has less antibacterial activity.

## 5. Conclusion

*S aureus* and *P. aeruginosa* cause mild to dreadful eye infections. The findings in our study would help the clinicians as well as the patients use contact lens multipurpose solutions which follow the guidelines of ISO 14729 standards as it reduces the chances of acquiring ocular infections among contact lens wearers. It is recommended that the manufacturers also follow the guidelines for the quality check of the solutions. The effectiveness of MPS varies against different bacterial species. Biotrue has shown good antimicrobial activity and disinfecting property as compared to Aquasoft and Opti-Free Replenish. The current study does not evaluate the efficacy of the solutions in clinical isolates; hence, variations may be observed. We have observed that the antimicrobial activity of different solutions varies with respect to time of incubation, and also there was a marked difference in the activity of some solutions against *S. aureus* and *P. aeruginosa*. The solution with better disinfecting action and sufficient hygiene measures is recommended for everyday use for cleaning by contact lens users.

## Figures and Tables

**Table 1:** Colony counts per solution per time interval for *Staphylococcus aureus*.

Time	Biotrue	Aquasoft	Opti-Free Replenish	Control: saline
Zero	100,000	100,000	100,000	100,000
10 minutes	33,400	12,000	37,200	100,000
30 minutes	9500	8800	11,600	100,000
Two hours	0	6200	2800	100,000
Four hours	0	0	2200	100,000
Six hours	0	0	1800	100,000

**Table 2:** Colony counts per solution per time interval for *Pseudomonas aeruginosa*.

Time	Biotrue	Aquasoft	Opti-Free Replenish	Control: saline
Zero	100,000	100,000	100,000	100,000
10 minutes	10,300	7000	10,000	100,000
30 minutes	1500	6400	8200	100,000
Two hours	400	1700	7800	100,000
Four hours	0	1100	6000	100,000
Six hours	0	0	5552	100,000

**Table 3:** Log reduction per solution per time interval for *Staphylococcus aureus.*

Time	Biotrue	Aquasoft	Opti-Free Replenish
10 minutes	0.48	0.93	0.43
30 minutes	1.02	1.06	0.94
Two hours	5	1.21	1.56
Four hours	5	5	1.66
Six hours	5	5	1.75

**Table 4:** Log reduction per solution per time interval for *Pseudomonas aeruginosa.*

Time	Biotrue	Aquasoft	Opti-Free Replenish
10 minutes	0.99	1.16	1
30 minutes	1.83	1.21	1.09
Two hours	2.40	1.77	1.11
Four hours	5	1.96	1.23
Six hours	5	5	1.26

## Data Availability

The data used to support the findings of this study are available from the corresponding author upon request.

## References

[1] Oberholzer M., Raubenheimer J., Lyell M. (2015). A comparison of the ability of three common contact lens solutions with different constituents to inhibit the growth of *Staphylococcus aureus*. *African Vision and Eye Health*.

[2] Amiri M., Mohammadinia M., Tabatabaee M. (2011). Comparative efficacies of contact lens disinfecting solutions against *Pseudomonas aeruginosa*. *Clinical and Experimental Optometry*.

[3] Mohammadinia M., Rahmani S., Eslami G. (2011). Contact lens disinfecting solutions antibacterial efficacy: comparison between clinical isolates and the standard ISO ATCC strains of *Pseudomonas aeruginosa* and *Staphylococcus aureus*. *Eye*.

[4] Chang D. C., Grant G. B., O’Donnell K. (2006). Multistate outbreak of Fusarium keratitis associated with use of a contact lens solution. *JAMA*.

[5] Miller M., Callahan D., McGrath D., Manchester R., Norton S. (2000). CL-160: disinfection efficacy of contact lens care solutions. *Optometry and Vision Science*.

[6] Manuj K., Gunderson C., Troupe J., Huber M. (2006). Efficacy of contact lens disinfecting solutions against *Staphylococcus aureus* and *Pseudomonas aeruginosa*. *Eye and Contact Lens: Science and Clinical Practice*.

[7] Demirbilek M., Evren E. (2014). Efficacy of multipurpose contact lens solutions against ESBL-positive *Escherichia coli*, MRSA, and *Candida albicans* clinical isolates. *Eye and Contact Lens: Science and Clinical Practice*.

[8] Lever A., Borazjani R. (2001). Comparative antimicrobial efficacy of multi-purpose hydrogel lens care solutions. *Contact Lens and Anterior Eye*.

[9] Iguban E., Nanagas J. (2016). The in vitro anti-microbial activity of multipurpose contact lens solutions against standard strains of common ocular pathogens: the effect of duration from first use. *Journal of Clinical and Experimental Ophthalmology*.

[10] Sakuma S., Reeh B., Dang D., Harris M. (1996). Comparative efficacies of four soft contact lens disinfection solutions. *International Contact Lens Clinic*.

[11] Kuzman T., Kutija M., Kordic R. (2013). Comparative study of antibacterial an antifungal effects of rigid gas permeable contact lens disinfecting solutions. *Collegium Antropologicum*.

[12] Hildebrandt C., Wagner D., Kohlmann T. (2012). In-vitro analysis of the microbicidal activity of 6 contact lens care solutions. *BMC Infectious Diseases*.

